# An ancient spliceosomal intron in the ribosomal protein L7a gene (*Rpl7a*) of *Giardia lamblia*

**DOI:** 10.1186/1471-2148-5-45

**Published:** 2005-08-18

**Authors:** Anthony G Russell, Timothy E Shutt, Russell F Watkins, Michael W Gray

**Affiliations:** 1 Program in Evolutionary Biology, Canadian Institute for Advanced Research, Department of Biochemistry and Molecular Biology, Dalhousie University, Halifax, Nova Scotia B3H 1X5, Canada

## Abstract

**Background:**

Only one spliceosomal-type intron has previously been identified in the unicellular eukaryotic parasite, *Giardia lamblia *(a diplomonad). This intron is only 35 nucleotides in length and is unusual in possessing a non-canonical 5' intron boundary sequence, CT, instead of GT.

**Results:**

We have identified a second spliceosomal-type intron in *G. lamblia*, in the ribosomal protein L7a gene (*Rpl7a*), that possesses a canonical GT 5' intron boundary sequence. A comparison of the two known *Giardia *intron sequences revealed extensive nucleotide identity at both the 5' and 3' intron boundaries, similar to the conserved sequence motifs recently identified at the boundaries of spliceosomal-type introns in *Trichomonas vaginalis *(a parabasalid). Based on these observations, we searched the partial *G. lamblia *genome sequence for these conserved features and identified a third spliceosomal intron, in an unassigned open reading frame. Our comprehensive analysis of the *Rpl7a *intron in other eukaryotic taxa demonstrates that it is evolutionarily conserved and is an ancient eukaryotic intron.

**Conclusion:**

An analysis of the phylogenetic distribution and properties of the *Rpl7a *intron suggests its utility as a phylogenetic marker to evaluate particular eukaryotic groupings. Additionally, analysis of the *G. lamblia *introns has provided further insight into some of the conserved and unique features possessed by the recently identified spliceosomal introns in related organisms such as *T. vaginalis *and *Carpediemonas membranifera*.

## Background

Spliceosomal introns have now been identified in all major eukaryotic lineages. Recently added to this list are several protists that are widely considered to represent deep divergences within Eucarya: the diplomonad *Giardia lamblia *[1], its close relative *Carpediemonas membranifera *[2], and the parabasalid *Trichomonas vaginalis *[3]. The distribution and conservation of proteins involved in the removal of spliceosomal introns [1,4,5] suggests that this intron type is a feature that was present in the ancestor of this domain of life. While the precise determination of intron frequency in the *T. vaginalis *and *C. membranifera *genomes awaits further analysis, these preliminary studies suggest that these two organisms likely contain many more spliceosomal introns than are evident in the compact genome of *G. lamblia*. To date, only one spliceosomal-type intron has been reported in *G. lamblia *despite the analysis of approximately 5800 predicted open reading frames (ORFs) [1].

One of the most interesting properties of the *T. vaginalis *introns is the presence of a functionally important motif and putative branch-point sequence, ACTAAC, invariantly located within a 'conserved' 12-nucleotide (nt) intron segment located directly adjacent to the 3' splice site. Surprisingly, the only known *G. lamblia *intron also contains the identical 12-nt motif at its 3' end. Additionally, the introns from these two organisms display significant sequence similarity at their 5' ends. This suggests that similar splicing mechanisms may be employed to remove introns in organisms from these two eukaryotic groups, members of the proposed eukaryotic supergroup Excavata [6]. These sequence motifs have been shown to be important for efficient *in vivo *splicing of introns in *T. vaginalis *[3]; however, since only one intron has been identified in *G. lamblia *so far, the extent of conservation of these sequence motifs in other *G. lamblia *introns is not known. It is conceivable that these intron features are indicative of ancestral eukaryotic spliceosomal introns. Conversely, these features may be derived and thus be unique among spliceosomal introns found in these long-branching eukaryotic taxa.

It has been proposed that most of the identified *T. vaginalis *introns are ancient because introns are also found at similar positions in homologous genes in some other eukaryotic taxa [3]. Since the taxonomic sampling addressing the prevalence of any of these introns is sparse and because many of these introns are not located in the same phase or even the same relative amino acid position, the actual conservation and ancient nature of these introns requires further verification. The possibility that a subset of spliceosomal introns could be ancestral to the eukaryotic radiation is an exciting one and, if true, suggests that these introns might be useful genomic markers to aid in the elucidation of deep phylogenetic relationships within the domain Eucarya.

Recently, hypotheses describing eukaryotic evolution have coalesced around a limited number of eukaryotic supergroups. One scheme [7] proposes six primary eukaryotic clades: Opisthokonta, Amoebozoa, Plantae, Chromalveolata [8], Rhizaria [9] and Excavata [6,10]. The strength of the evidence supporting each of these supergroups varies, as does the degree of organismal sampling within the proposed assemblages. The relationships among the supergroups themselves are currently undefined: i.e., their relative branching order in the eukaryotic tree is unknown. Even with concatenated data sets comprising hundreds of individual sequences, the approaches of molecular phylogenetics are increasingly challenged to provide robust and compelling answers to this question.

In this report, we identify an intron in *Rpl7a*, the gene encoding the *G. lamblia *ribosomal protein L7a and an additional spliceosomal intron in an unassigned ORF that encodes a non-conserved protein. We observe striking similarities among the *G. lamblia*, *T. vaginalis *and *C. membranifera *introns. At the same time, departures from the sequence constraints within these motifs may discriminate some of the splicing mechanisms employed within these eukaryotic groups. Our extensive examination of the phylogenetic distribution and properties of the *Rpl7a *intron indicate that it is an ancient spliceosomal intron, and our study also provides preliminary evidence uniting three eukaryotic supergroups (Opisthokonta, Amoebozoa, Excavata) to the exclusion of at least two others (Chromalveolata, Plantae). Our investigation of the distribution of the *Rpl7a *intron is the most extensive examination to date of a conserved intron position in eukaryotes. Taken together, these results argue that further examination of the patterns of intron conservation and distribution within the eukaryotic domain, like other shared derived characters such as gene fusions, insertions and gene replacements, can provide a valuable adjunct for evaluating proposed phylogenetic groupings and frameworks derived from sequence comparisons.

## Results and discussion

### A conventional spliceosomal intron in the *Giardia lamblia Rpl7a *gene

**Figure 1:**
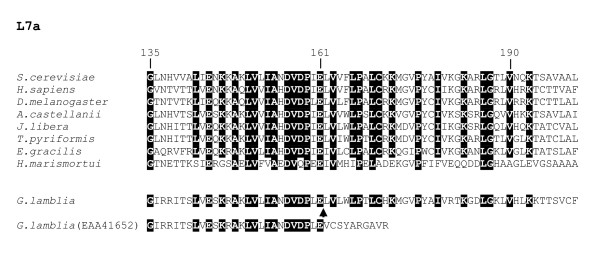
**Clustal X alignment of ribosomal protein L7a amino acid sequences. **The alignment comprises the portion corresponding to positions 135 to 200 in the *Saccharomyces cerevisiae *protein (full organism names are listed in Table 1). At highly conserved positions at which a single amino acid predominates, residues are indicated as white letters on a black background. The arrowhead denotes the location of a conserved spliceosomal intron. The predicted *Giardia lamblia *protein sequence either with (above arrowhead) or without (below arrowhead) removal of the intron is shown. Sources of the sequences are: *S. cerevisiae *[GenBank:AAB65045], *Homo sapiens *[GenBank:NP_000963], *D. melanogaster *[GenBank:AAN09172], *A. castellanii *[GenBank:AY925000], *J. libera *[GenBank:AY924997], *T. pyriformis *[GenBank:DQ118092], *E. gracilis *[GenBank:], *H. marismortui *[GenBank:YP_134885] and *G. lamblia *[GenBank:AACB01000019].

**Figure 2:**
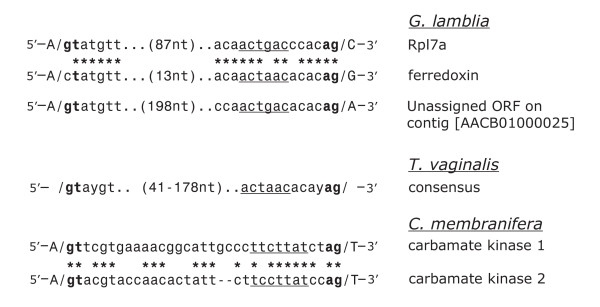
**Conservation of intron boundary sequences in *Giardia lamblia*, *Trichomonas vaginalis *and *Carpediemonas membranifera*. **Positions of nucleotide identity within the intron sequences in *G. lamblia *and *C. membranifera *are indicated with asterisks. Potential intron branch-point sequences are underlined. The distance between the intron boundary sequences is indicated for each *G. lamblia *intron as is the distance variation between these sequences seen in the *T. vaginalis *introns used to derive the consensus sequence shown. The ferredoxin intron sequence is from [1], the *T. vaginalis *introns are from [3], and the carbamate kinase intron sequences are from [2].

In comparing ribosomal protein L7a homologs from various organisms, we noted that the predicted sequence for the *G. lamblia *protein [GenBank:EAA41652] appeared abnormally truncated at the C-terminal end relative to other eukaryotic and archaeal sequences, terminating at position 171 of *S. cerevisiae *L7a (Fig. 1). In addition, this truncated sequence displays unusual divergence after yeast position 161, which is unexpected because this C-terminal region corresponds to a highly conserved portion of L7a. Further examination of the *G. lamblia *gene sequence revealed a 109-nt intron whose removal results in a predicted L7a protein sequence similar in length to that of other eukaryotic L7a homologs, and that aligns readily with them downstream of position 161 (Fig. 1). The assigned intron boundary sequences, GT...AG (Fig. 2), are those of conventional spliceosomal introns.

The *Rpl7a *intron is only the second reported spliceosomal-type intron in *G. lamblia*, a diplomonad widely considered to be a deep-branching eukaryote. The other example (35 nt in length) resides in a putative [2Fe-2S] ferredoxin gene and begins with CT rather than GT at the 5' intron boundary [1]. Comparison of the sequences of the two *G. lamblia *introns reveals striking sequence conservation at the intron termini (Fig. 2). Notably, this similarity includes the region predicted to contain the intron branch-point sequence [1] (underlined in Fig. 2). Likewise, both the 5' and 3' intron boundary sequences of the two *G. lamblia *introns display extensive sequence similarity to the consensus sequence of the known *T. vaginalis *introns (Fig. 2). However, there are some significant nucleotide differences between the *G. lamblia Rpl7a *intron and the *T. vaginalis *consensus motif that suggest somewhat different sequence requirements for splicing in these two organisms.

As mentioned previously, both CT and GT sequences have now been observed at the 5' intron boundary of *G. lamblia *introns. This observation is made even more significant by the demonstration of efficient splicing of the *G. lamblia *ferredoxin intron in a *T. vaginalis in vivo *splicing system, but only after changing the CT to a canonical GT at the intron 5' boundary [3]. Our identification of a second *G. lamblia *intron that contains a canonical GT indicates that the CT sequence is not an absolute requirement for splicing of these introns in *G. lamblia *and also suggests that the constraints on 5' intron boundary sequences are different in these two organisms.

The second important observation is derived from a comparison of the sequences of the putative branch-point motifs contained within the two *G. lamblia *introns. The *G. lamblia *ferredoxin intron has the putative branch-point sequence ACTAAC, which is identical to that observed in all the identified *T. vaginalis *introns. The *G. lamblia Rpl7a *intron instead contains the sequence ACTGAC. By creating site-directed mutants of a *T. vaginalis *splicing reporter construct using an intron from a poly(A) polymerase gene, Vaňáčová *et al*. [3] demonstrated the importance of this sequence element and its position relative to the intron 3' end for efficient *in vivo *splicing in *T. vaginalis*. However, single-nucleotide substitutions at the position corresponding to the G nucleotide in the branch-point sequence of the *G. lamblia Rpl7a *intron were not examined for their effects on splicing. Therefore, it is possible that ACTGAC could be a functional branch-point sequence for the splicing of *T. vaginalis *introns.

The majority of the introns identified in the *T. vaginalis *genome were found using a sequence-pattern search strategy based primarily on nucleotide conservation observed between the intron boundary sequences of the first identified *T. vaginalis *intron and the *G. lamblia *ferredoxin intron. The identified introns, 39 in total, vary in length between 59 and 196 nt. They contain a consensus 5' splice site sequence GTAYGT and, based on constraints imposed in the search parameters, the 3' splice-site sequence ***ACTAAC***ACAYAG, where the nucleotides in bold italics indicate the potential branch-point sequence.

To investigate whether an additional group of *T. vaginalis *introns might instead contain variations at the fourth nucleotide position (bold italics) of the branch-point sequence, ACT***A***AC, the partial *T. vaginalis *genome sequence was searched for potential introns containing these properties. We also incorporated into our search parameters an allowance for other sequence differences within the 3' intron boundary sequence, such as those observed when comparing the two *G. lamblia *introns. Using the pattern-search algorithm PatScan [11], we searched the entire preliminary genomic data (3X coverage) for all sequences containing the sequence pattern [5'– GTAYGT...5–500 nt...***ACTBAC***NCAYAG-3'], where B = C, G or T; Y = C or T; N = any nucleotide and the branch-point sequence is in bold italics. Remarkably, only two matches to this pattern were found in the entire *T. vaginalis *genome data set and neither of these sequence patterns appears to be an intron candidate. Furthermore, neither of these two sequences contains ACTGAC in the predicted position for the intron branch-point sequence. This result further emphasizes the importance of the ACTAAC sequence motif in the *T. vaginalis *introns and the differences in sequence constraints apparent between these introns and those of *G. lamblia*. If additional *T. vaginalis *spliceosomal introns are found (<519 nt in size) that contain alternative sequences at the 3' intron boundary, sequence differences must also be present in the 5' intron boundary that prevent the search algorithm from finding these intron candidates using the parameters we have employed.

Using search parameters similar to those above (but allowing G or C as the starting nucleotide at the intron 5' end), we searched the *G. lamblia *partial genomic data set for intron candidates. As expected, the *Rpl7a *and ferredoxin introns are detected by this method, as is an additional set of 15 matches to this sequence pattern. Of these matches, only one was a likely intron candidate that had the potential to disrupt a predicted ORF, among other criteria. Since the ORF in which this candidate intron resides (or that it disrupts) does not encode a conserved protein – unlike the *Rpl7a *intron – further experimentation was required to prove whether or not it is an intron. Removal of the intron candidate sequence, nt positions 1263 to 1482 of the *G. lamblia *contig [GenBank:AACB01000025], extends the predicted protein encoded by the unassigned ORF [GenBank:EAA41257] by an additional 118 amino acids at its amino terminus. Further, when the intron sequence is removed, the inferred initiation codon is now positioned directly adjacent to an AT-rich motif that Yee *et al. *[12] have previously identified as a promoter element for other *G. lamblia *genes. RT-PCR analysis and sequencing of cDNA clones obtained from the corresponding mRNA (see Additional File 1) confirm the existence of this third *G. lamblia *spliceosomal intron, which is 220 nt long and exhibits intron boundary sequences similar to those of the ferrodoxin and *Rpl7a *introns (Fig. 2).

Extensive genomic DNA information is not yet available for *C. membranifera*, and to date only two introns, both small, have been identified in this protist [2]. In these introns, potential branch-point sequences (underlined in Fig. 2) are found abutting the 3' intron boundary, as is observed in the *G. lamblia *and *T. vaginalis *introns. While the *G. lamblia *introns lack an obvious polypyrimidine tract in the vicinity of the 3' end of the intron, *C. membranifera *introns and the majority of the *T. vaginalis *introns do exhibit pyrimidine-rich sequences immediately upstream of the 3' intron/exon boundary. However, in the *C. membranifera *introns these pyrimidine-rich sequences include the potential branch-point sequences whereas in *T. vaginalis *these sequences (usually T-rich) are found upstream of the potential branch-point sequences. It is also apparent that the *C. membranifera *introns exhibit less sequence conservation at their 3' intron boundaries than do the *G. lamblia *and *T. vaginalis *introns. These important differences in the properties apparent in *C. membranifera *introns are particularly relevant to this comparison of intron structure because this organism appears to have a closer evolutionary relationship to *G. lamblia *than does *T. vaginalis *[13]. The variety of differences observed in intron structure from each of these three organisms seems to further differentiate the sequence requirements for the splicing mechanisms employed in these eukaryotic groups. The identification of homologs of snRNAs in these organisms may give insight and permit comparisons of novel splicing mechanisms employed in the removal of these introns, characterized by the above-mentioned conserved intronic elements and potential branch-point sequences unusually close to the intron 3' end.

### Phylogenetic distribution and properties of the *Rpl7a *intron indicate that it is ancient

**Figure 3:**
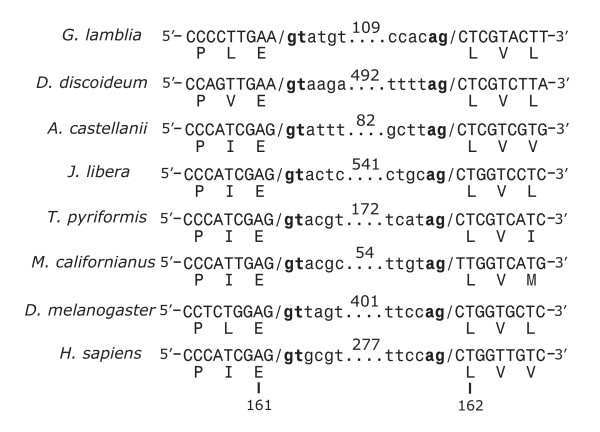
**An evolutionarily conserved spliceosomal intron within *Rpl7a *of representative organisms of the domain Eucarya. **Nucleotide sequences at the exon-intron junctions are shown, with the exon sequences in uppercase letters and intron sequences in lowercase. The total length of each intron is shown above the dotted lines. The conventional spliceosomal intronic boundary sequences are bolded (**gt**...**ag**). One-letter amino acid abbreviations for the corresponding L7a protein sequences are indicated below the exon sequences. Amino acid position within the L7a protein is indicated at the bottom of the figure and corresponds to the *S. cerevisiae *sequence, as in Fig. [1]. Sequences from NCBI are: *D. discoideum *[GenBank:AC116100], *D. melanogaster *[GenBank:X82782]; *H. sapiens *[GenBank:X52138]; and *G. lamblia *[GenBank:AACB01000019]. Sequences determined in the present study are: *A. castellanii *[GenBank:AY925008]; *J. libera *[GenBank:AY925006]; *T. pyriformis *[GenBank:AY925011]; *M. californianus *[GenBank:AY925003].

Given the phylogenetic conservation of the L7a protein in the eukaryotic domain, we searched available eukaryotic genomic databases to determine whether an intron at the same position as in *G. lamblia *is conserved in the *Rpl7a *gene of other organisms. Somewhat surprisingly, we identified a conventional spliceosomal intron at exactly the same position and in the same phase within *Rpl7a *in many animal (opisthokont) taxa and in the amoebozoon *Dictyostelium discoideum *(Fig. 3). This result, in concert with the proposed deep branching position of *Giardia*, suggested that the *Rpl7a *intron might be an ancient eukaryotic spliceosomal intron. Accordingly, we undertook a more detailed investigation of the occurrence of this particular intron within the eukaryotic domain. Using EST sequences available through the Protist EST Program (PEP), PCR primers were designed to amplify genomic *Rpl7a *sequences from a wider array of eukaryotes, particularly those placed in the controversial taxon Excavata [14]. We identified the *Rpl7a *intron in three additional Amoebozoa representatives spanning amoebozoan diversity [15]. We also found the intron in three additional groups from Excavata: jakobids, malawimonads and *Trimastix pyriformis *(Fig. 3). The intron is variable in size in these organisms but was only ever found in frame 0 of the coding region. The jakobid *Rpl7a *introns (541–988 nt) are the largest characterized to date in this taxon, notably longer than the 156-nt intron found in the *J. libera β*-tubulin gene [16]. The *Rpl7a *intron is also the first reported example of an intron from *Trimastix*, although other introns have been identified in this organism (A.J. Roger, pers. comm.). *Malawimonas jakobiformis *appears to have two copies of *Rpl7a*, each containing the intron. Table 1 summarizes the organisms surveyed for the presence of the *Rpl7a *intron.

**Table 1:** Organisms surveyed for the presence of the *Rpl7a *intron

**Organism**^1^	**Number**^2^	**Intron Size (nt)**
		
**With intron:**		
		
***Opisthokonta (Metazoa only)***		
Vertebrata	(7)	
*Homo sapiens*^†^		277
Chordata	(2)	
*Ciona intestinalis*^†^		586
Hexapoda	(5)	
*Drosophila melanogaster*^†^		401
		
***Amoebozoa***		
*Dictyostelium discoideum*^†^		492
Physarum polycephalum*^†^		103
Acanthamoeba castellanii*^†^		82
Hartmannella vermiformis*^†^		58
		
***Excavata***		
Jakobidae		
Jakoba libera*^†^		541
Reclinomonas americana*^†^		877
Seculamonas ecuadoriensis*^†^		988
Trimastix (Trimastix pyriformis*^†^)		172
Malawimonadidae		
Malawimonas jakobiformis*^†^		53, 55^3^
Malawimonas californianus*^†^		54
Diplomonadida (*Giardia lamblia*)		109
		
**Without intron:**		
		
***Chromalveolata***		
Alveolata	(14)	
Heterokonta (stramenopiles)	(3)	
		
***Plantae***		
Streptophyta	(5)	
Rhodophyta	(2)	
Chlorophyta	(1)	
		
***Rhizaria***		
Cercozoa (Bigelowiella natans*)	(1)	
		
***Opisthokonta***		
Fungi	(28)	
Nematoda/Trematoda	(4)	
Hexapoda	(1)	
Capsaspora owczarzaki*		
		
***Amoebozoa***		
Entamoebidae	(4)	
		
***Excavata***		
Heterolobosea (Naegleria gruberi*)		
Euglenozoa		
Trypanosomatidae	(7)	
Euglenida (Euglena gracilis*)		
Parabasalia (*Trichomonas vaginalis*)		
Diplomonadida (Spironucleus barkhanus*)		

^1^An asterisk (*) denotes organisms for which PCR amplification of the *Rpl7a *gene was performed; ^† ^indicates that expressed sequence tag (EST) data exist that confirm the absence of the intron in the corresponding mRNA.

^2^Numbers in brackets refer to the number of organisms examined within each group; a complete list is provided in Additional File 3.

^3^Two versions of the *Rpl7a *gene exist in this organism, containing introns of slightly different length.

Within Opisthokonta and Amoebozoa, several groups appear to lack the *Rpl7a *intron. Over time introns are randomly lost, so a punctate distribution within established eukaryotic groups is not unexpected. Independent cases of intron loss can be inferred when the relationship among groups is known and the intron is present in a common ancestor. This is the case for taxa from the Opisthokonta and the Entamoebidae (amoebozoons) that are missing the *Rpl7a *intron. The *Rpl7a *intron is present in several amoebozoons that branch outside [15,17] of those that lack the intron. A particularly interesting case of predicted intron loss is the *Anopheles gambiae Rpl7a *[GenBank:AAAB01008960], which lacks any introns. This situation is in stark contrast to other hexapod *Rpl7a *sequences, which contain introns in addition to the conserved intron discussed here. The *A. gambiae *case may be an example of intron loss mediated by a reverse transcription mechanism [18,19] and is also consistent with observed intron loss patterns in the hexapods [20].

None of the examined members of Plantae, Chromalveolata or Rhizaria contained the conserved *Rpl7a *intron. Only a single member of Rhizaria (*B. natans*) was investigated here so it is premature to conclude that the *Rpl7a *intron is absent altogether from this supergroup. Our survey failed to detect the *Rpl7a *intron in representatives of the Euglenozoa, Heterolobosea or Parabasalia, all eukaryotic groups for which inclusion in a larger Excavata assemblage is only weakly supported [6]. Whereas emerging data suggest that *E. gracilis *is well endowed with spliceosomal introns [21-23], intron distribution appears to be sparse in heteroloboseans [24]. The diplomonad *Spironucleus barkhanus *does not have the *Rpl7a *intron, likely a result of intron loss in this organism given the presence of the intron in other related Excavata.

We considered other (less probable) explanations that the phylogenetic distribution of the *Rpl7a *intron could be explained by independent events of intron gain or by lateral gene transfers. We note that the *Rpl7a *exon boundary sequences conform poorly to the conserved proto-splice site sequence (A,C)AG/G [25] (Fig. 1 and 3). The predicted amino acids flanking the intron insertion site are highly conserved, including in the archaeal homologs, resulting in a functional constraint on the DNA sequence abutting the intron. This observation argues against a distribution of the *Rpl7a *intron resulting from multiple intron gains at proto-splice sites. Supporting the argument against intron gain is the apparent very low density of introns in *G. lamblia*. This is also in agreement with recent data suggesting that few shared intron positions between distantly related taxa are due to parallel gain (i.e., independent insertion) at proto-splice sites [25].

The additional possibility exists that the phylogenetic distribution of the intron, particularly its presence in *G. lamblia*, could reflect eukaryote-to-eukaryote lateral gene transfer events. A phylogenetic analysis (see Additional File 2) of L7a protein sequences from 20 representative eukaryotes and two archaeons does not indicate an obvious unexpected affinity of *G. lamblia *with any other eukaryotic group. Cumulatively, the above observations argue that the *Rpl7a *intron is ancestral to many eukaryotic groupings and has been lost sporadically in various eukaryotic taxa, in which case the *Rpl7a *intron would be one of the oldest introns found to date.

### Phylogenetic implications

Depending on one's views of the eukaryotic tree, there are two possibilities to explain the observed distribution of the *Rpl7a *intron with regard to intron loss. In the context of more recent proposals of a basally unresolved eukaryotic tree [7,26], the *Rpl7a *intron would have to have been lost multiple times at the base of Plantae, Chromalveolata and (tentatively) Rhizaria, but maintained in Opisthokonta, Amoebozoa and Excavata. Alternatively, if one assumes a specific relationship among Opisthokonta, Amoebozoa and Excavata, it is only necessary to invoke a single loss of the *Rpl7a *intron in a common ancestor of Plantae/Chromalveolata/Rhizaria, to explain the apparent absence of this intron in these three supergroups.

While multiple cases of intron gain seem unlikely (as discussed above), a single intron gain at the base of Opisthokonta, Amoebozoa and Excavata would result in the observed distribution, assuming the Plantae/Chromalveolata/Rhizaria had already diverged. While additional intermediate possibilities exist with regards to various intron loss and/or gain events, we propose that a single loss or gain is the most parsimonious explanation for the observed distribution of the *Rpl7a *intron, supporting a grouping of Opisthokonta, Amoebozoa and Excavata.

Although the distribution of the *Rpl7a *intron does not position the root of the eukaryotic tree, it may help to resolve some of the basal branches. It is important to note that Excavata is a tenuous grouping that may in fact be polyphyletic: thus, members of Excavata without the intron may fall on either side of a putative loss/gain event. In this instance, it is possible that one or more members of Excavata will be found to group with the Plantae/Chromalveolata/Rhizaria consortium. Conversely, if additional evidence robustly groups the organisms in question within Excavata, this result would imply that they lost the intron independently, as is evidently the case for Fungi within Opisthokonta.

New eukaryotic genome sequences may well reveal the *Rpl7a *intron in other representatives of Plantae, Chromalveolata and/or (particularly) Rhizaria than those listed in Table 1. Although such a finding would necessarily require reinterpretation of some of the conclusions reached here, discovery of the *Rpl7a *intron in these other eukaryotic supergroups would only strengthen the argument that this intron is indeed ancient. At present, the simplest explanation for the observed distribution of the *Rpl7a *intron is a specific relationship uniting Opisthokonta, Amoebozoa and Excavata to the exclusion of Plantae, Chromalveolata and possibly Rhizaria. However, additional characters will need to be found in order to strengthen this proposed assemblage.

## Conclusion

The properties possessed by the first identified *G. lamblia *spliceosomal-type intron (35 nt in length) raised questions regarding the importance of its non-canonical 5' intron boundary and possible constraints on the size of *Giardia *introns. Furthermore, it was clear that the identification of additional spliceosomal introns would be required to assess the degree of conservation of predicted functional sequence elements within these introns [27]. In this study we have identified in *G. lamblia *two larger introns (109 and 220 nt) in genes encoding, respectively, L7a and a non-conserved, unassigned protein. Both of these newly identified introns exhibit a canonical GT 5' intron boundary. Evolutionary conservation of the exact position and phase of the *Rpl7a *intron (within a highly conserved region of the L7a protein sequence in members of diverse eukaryotic groups) indicates that this particular intron was likely present in the ancestor of these lineages. The *Rpl7a *intron exhibits all of the hallmark features of an ancient intron, such as widespread distribution, phase 0 positioning, location in an ancient eukaryotic gene, and lack of a proto-splice site sequence; thus, this intron can be considered to be a meaningful phylogenetic marker. Currently, no singular genomic marker definitively resolves the branching order of the eukaryotic supergroups. We propose that patterns of conservation of ancient introns, when larger data sets are examined, may provide such information.

## Methods

### Genomic DNA

Samples of genomic DNA from various protists (full organism names are listed in Table 1) were kindly provided by B.F. Lang (*R. americana*, ATCC 50394; *S. ecuadoriensis*, ATCC 50688; *M. jakobiformis*, ATCC 50310; *M. californianus*, ATCC 50740); A.J. Roger (*S. barkhanus *strain NOR-1A, ATCC 50380+; *C. owczarzaki*, ATCC 30864; *T. pyriformis*, ATCC 50562; *N. gruberi*, ATCC 30224); A.J. Lohan (*A. castellanii*, ATCC 30010; *H. vermiformis*, ATCC 50236); D.F. Spencer (*E. gracilis *strain Z); and J.M. Archibald (*B. natans*, CCMP 621). Genomic DNA from *J. libera *(ATCC 50422) and *P. polycephalum *(strain M_3_C) was obtained by lysing cells in 1% SDS followed by phenol extraction and ethanol precipitation.

### Characterization of *Rpl7a *sequences

Polymerase chain reaction (PCR) was used to amplify *Rpl7a *from genomic DNA (100–200 ng), using Invitrogen Taq DNA polymerase. PCR primers used are listed in Additional file 3: Supplemental Tables. PCR cycling conditions were: 3 min at 95°C; 35 cycles of 30 sec at 95°C, 30 sec at 55°, 1 min at 72°C; and 10 min at 72°C. PCR product bands were isolated from gels using the Sephaglas™ BandPrep Kit (Amersham Pharmacia Biotech) and the recovered DNA was cloned into the pCR® 2.1-TOPO® vector using the TOPO TA Cloning® Kit (Invitrogen). DNA sequencing was performed using an automated ABI Prism 377 DNA sequencer.

### Computer analyses

Ribosomal protein L7a gene and protein sequences were identified by searching relevant sequence databases using TBLASTN or BLASTP with the *S. cerevisiae *sequences as queries. Protein alignments were generated with ClustalX 1.82 [28] applying default alignment parameters. DNASIS V 2.5 was used for translation of DNA sequences and to assist in the identification of introns within *Rpl7a *sequences. Searches for spliceosomal-like introns within the preliminary genome sequence data of *T. vaginalis *and *G. lamblia *were performed using the sequence pattern search program PatScan [11], recompiled with the constant 'MAX_SEQ-LEN' redefined to '100000000'.

## Authors' contributions

AGR analyzed the L7a protein sequences, discovered the *G. lamblia *Rpl7a intron, performed genomic searches for additional introns in *G. lamblia *and *T. vaginalis *and carried out the RT-PCR experiments to confirm the presence of the third *G. lamblia *spliceosomal intron. TES examined the phylogenetic conservation of the *Rpl7a *intron in currently available eukaryotic genome databases, performed all the PCR experiments to determine the prevalence of the intron, and participated in the genomic searches for additional *G. lamblia *introns. RFW performed the phylogenetic analysis of L7a sequences. MWG made substantial intellectual contributions to this work. All authors participated in the assembly and editing of the manuscript. All authors read and approved the final manuscript.

## Supplementary Material

Additional File 1 **Evidence for a spliceosomal intron in an unassigned *G. lamblia *ORF. **This file (PDF format) presents gel electrophoretic data documenting the results of PCR and RT-PCR experiments to confirm the existence of a putative spliceosomal intron in a non-conserved, unassigned ORF in *G. lamblia*, as described in the text. Click here for file

Additional File 3 **Supplemental Tables. **This file (PDF format) contains two tables. Table 1 contains the sequences of the oligonucleotide primers used for amplifying *Rpl7a *sequences from various eukaryotic taxa. Table 2 is a complete list of all the organisms and sequence sources that were examined for the presence of the *Rpl7a *intron. Click here for file

Additional File 2 **L7aTree. **This file (PDF format) presents a maximum likelihood tree generated using L7a sequences from representative eukaryotic and archaeal taxa. Also included are the methods used to generate the tree, the sources of the sequences and the alignment that was used. Click here for file
