# Supplementary material for: An ancient spliceosomal intron in the ribosomal protein L7a gene (Rpl7a) of Giardia lamblia
Source: BMC Evol Biol. 2005 Aug 18;5:45. doi: 10.1186/1471-2148-5-45 (PMC1201135; doi:10.1186/1471-2148-5-45)
Supplement: Additional File 1 — Evidence for a spliceosomal intron in an unassigned G. lamblia ORF. This file (PDF format) presents gel electrophoretic data documenting the results of PCR and RT-PCR experiments to confirm the existence of a putative spliceosomal intron in a non-conserved, unassigned ORF in G. lamblia, as described in the text. [file 1471-2148-5-45-S1.pdf]

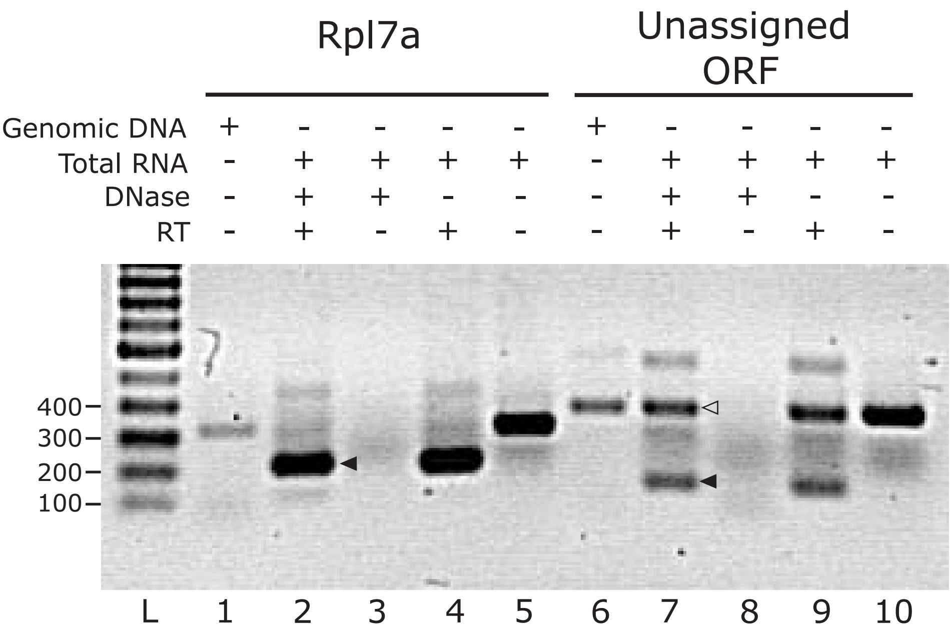

Rpl7a
Unassigned ORF
Genomic DNA + - - - - + - - - -
Total RNA - + + + + - + + + +
DNase - + + - - - + + - -
RT - + - + - - + - + -
400
300
200
100
L 1 2 3 4 5 6 7 8 9 10

Additional File 1: Evidence for a spliceosomal intron in an unassigned *G. lamblia* ORF.

Reverse-transcription polymerase chain reaction (RT-PCR) was performed to determine whether a predicted ORF of unknown function, whose sequence was initially identified within *G. lamblia* contig [Genbank AACB01000025], contains a spliceosomal intron. Detection of mature *Rpl7a* mRNA resulting from removal of the *Rpl7a* intron from its precursor mRNA served as a positive control. PCR reactions were also performed using 20 ng of *G. lamblia* genomic DNA as template (lanes 1 and 6). These lanes show the size of the products generated from the intron-containing genes and therefore the sizes expected for the RT-PCR products corresponding to unspliced precursor transcripts. RT-PCR was performed using 5 μg of *G. lamblia* total RNA, either with (lanes 2, 3, 7, 8) or without (lanes 4, 5, 9, 10) pre-treatment with DNase I, as the template in each cDNA synthesis reaction. The cDNA products corresponding to spliced mRNAs are indicated by black triangles whereas cDNA representing unspliced precursor is denoted with an open triangle. The cDNA and genomic products were cloned and sequenced to verify their identities, which also confirmed the predicted splice boundaries. Oligonucleotides used were oAR324 (5′-GCAGACGCTGGTCGTCTTCTTCA-3′), oAR325 (5′-GACAAGCTTGTCATCGCGTCTGG-3′), oAR330 (5′-CACGAGTAACGGTGTTTTCCTTC-3′) and oAR331 (5′-GACAAAATATGTTAATTTGTCCA-3′). Reverse transcription was performed on *G. lamblia* total RNA (a kind gift from Janet Yee, Trent University) using oligonucleotides oAR324 (complementary to positions 84116 to 84138 of *G. lamblia* contig AACB01000019) and oAR331 (complementary to 1535 to 1557 of contig

AACB01000025) as primers for *Rpl7a* and the unassigned ORF, respectively. In each annealing reaction, 5 µg of RNA was incubated with 2 pmol RT-primer for 5 min at 65ºC, 10 min at 47ºC followed by room temperature for 10 min. Reverse transcription was then performed using Superscript II RT (Invitrogen) according to the manufacturer's specifications, with incubation at 47ºC for 1 hr. The reaction mix was then heated to 90ºC for 5 min, following which ¼ of the RT reaction product was used as template for PCR with Taq DNA polymerase under standard PCR conditions. The portion of L7a coding sequence flanking the intron was amplified using oAR324 and oAR325. Likewise, the unassigned ORF was amplified using oAR330 (anneals to positions 1187 to 1209 of AACB01000025) and oAR331. Cycling parameters used were: 94ºC for 5 min, 35 cycles of 94ºC for 30 sec, 55ºC for 30 sec, 72ºC for 30 sec, followed by 72ºC for 7 min. PCR products were then separated by 1% agarose gel electrophoresis and visualized by ethidium bromide staining. Bands of interest were excised from the gel, purified using the Sephaglas™ BandPrep Kit (Amersham Pharmacia Biotech) and cloned into the pCR®2.1-TOPO® vector. DNA sequences were obtained using an automated Beckman Coulter CEQ™ 8000 sequencer.
