# Supplementary material for: An ancient spliceosomal intron in the ribosomal protein L7a gene (Rpl7a) of Giardia lamblia
Source: BMC Evol Biol. 2005 Aug 18;5:45. doi: 10.1186/1471-2148-5-45 (PMC1201135; doi:10.1186/1471-2148-5-45)
Supplement: Additional File 3 — Supplemental Tables. This file (PDF format) contains two tables. Table 1 contains the sequences of the oligonucleotide primers used for amplifying Rpl7a sequences from various eukaryotic taxa. Table 2 is a complete list of all the organisms and sequence sources that were examined for the presence of the Rpl7a intron. [file 1471-2148-5-45-S3.pdf]

Supplementary Table 1. Oligonucleotide primers used for genomic PCR spanning the region containing the conserved *Rpl7a* intron in organisms investigated in this study.

Organism	Forward Primer (5′-3′)	Reverse Primer (5′-3′)
A. castellanii	CCTACTTCGTGAAGTACGGCC	GGTCAAGAGCCTTACTGGTTGG
B. natans	GCGAAAGGACGCAGGGTTGC	CCTTCATCTTACGGCAGAGGG
C. owczarzaki	GGCTGAGGCTGTTGCCAAGG	GGACTTCTGGCCGAGAACACC
E. gracilis	GCCAGAATCCTCTTTCGAAAGGC	CGCATTGGCAGCAGCAATCC
H. vermiformis	GCCCAAGTTTGTCAAGATGGG	GCTCTTTCTTGGCCTCCTCC
J. libera	CCAGTTCCGCTTCACCCTGG	GAGTGCTCGTCGGCAACGTCG
M. jakobiformis	GCCGCTACGTTAAGTGGCCC	CGTACGGAACGTTGCGCTTGC
M. californianus	GCACAAGTACCGCCCTGAGG	GGATGTCCTCATAGCGGTCG
N. gruberi	GGCCAGTTTATGTCCGTCTCC	CGAAAGCACCTCTGACGGCTTC
P. polycephalum	GCAGCGCCTCAAGAAGTTGG	GCCTTGGGTCCCAACTTGCC
R. americana	GGACTTGACCCGCTTTGTGC	GCCCTTGACAATGCAGTACG
S. barkhanus	CGTGAAGAGCGTAAAGTTCG	GGGGAGCTTACGCTGAGACC
S. ecuadoriensis	CCAAGCTCGTGGTCATTGCC	GCCCTTGACAATGCAGTACG
T. pyriformis	GGCCAAGAAGGAGGGCAAGC	GCGCTGCTTCTCGTCCTTGG

Supplementary Table 2: Complete list of organisms investigated in this study for the presence of the *Rpl7a* intron.

**Organisms with intron**	**Website searched**
**Metazoa**	
Vertebrata	
Danio rerio	Sanger
Gallus gallus	Sanger
Homo sapiens	NCBI
Mus musculus	NCBI
Takifugu rubripes	NCBI
Tetraodon nigroviridis	NCBI
Xenopus laevis	NCBI
Chordata	
Ciona intestinalis	JGI
Ciona savignyi	NCBI
Hexapoda	
Apis mellifera	Sanger
Bombyx mori	NCBI
Drosophila melanogaster	NCBI
Drosophila pseudoobscura	NCBI
Drosophila yakuba	NCBI
**Amoebozoa**	
Acanthamoeba castellanii	PEPdb
Dictyostelium discoideum	NCBI
Hartmannella vermiformis	PEPdb
Physarum polycephalum	PEPdb
**Excavata**	
Jakobidae	
Jakoba libera	PEPdb
Reclinomonas americana	PEPdb
Seculamonas ecuadoriensis	PEPdb

Trimastix	
Trimastix pyriformis	PEPdb
Malawimonadidae	
Malawimonas californianus	PEPdb
Malawimonas jakobiformis	PEPdb
Diplomonadida	
Giardia lamblia	NCBI

**Organisms without intron**	**Website Searched**
**Chromalveolata**	
Alveolata	
Cryptosporidium hominis	http://cryptodb.org/blast/CryptoDBblast.shtml
Cryptosporidium parvum	http://cryptodb.org/blast/CryptoDBblast.shtml
Eimeria tenella	Sanger
Paramecium tetraurelia	http://paramecium.cgm.cnrs-gif.fr/ptblast/
Perkinsus marinus	TIGR
Plasmodium berghei	Sanger
Plasmodium chabaudi	Sanger
Plasmodium falciparum	NCBI
Plasmodium gallinaceum	Sanger
Plasmodium knowlesi	Sanger
Plasmodium reichenowi	Sanger
Tetrahymena thermophila	TIGR
Theileria parva	TIGR
Toxoplasma gondii	TIGR
Heterokonta (stramenopiles)	
Phytophthora ramorum	JGI
Phytophthora sojae	JGI
Thalassiosira pseudonana	JGI
**Plantae**	
Streptophyta	
Arabidopsis thaliana	TIGR
Oryza sativa	TIGR

Populus trichocarpa	JGI
Triticum aestivum	TIGR
Zea mays	TIGR
Rhodophyta	
Cyanidioschyzon merolae	http://merolae.biol.s.u-tokyo.ac.jp/blast/blast.html
Galdieria sulphuraria	http://genomics.msu.edu/galdieria/references.html
Chlorophyta	
Chlamydomonas reinhardtii	JGI
**Rhizaria**	
Cercozoa	
Bigelowiella natans	PEPdb
**Opisthokonta**	
Fungi	
Aspergillus fumigatus	NCBI
Aspergillus nidulans	NCBI
Candida albicans	NCBI
Candida glabrata	NCBI
Candida dubliniensis	Sanger
Coccidioides immitis	NCBI
Coccidioides posadasii	NCBI
Coprinopsis cinerea	NCBI
Cryptococcus neoformans var. grubii H99	NCBI
Cryptococcus neoformans var. neoformans B-3501A	NCBI
Cryptococcus neoformans var. neoformans JEC21	NCBI
Debaryomyces hansenii	NCBI
Encephalitozoon cuniculi	NCBI
Eremothecium gossypii	NCBI
Gibberella zeae	NCBI
Kluyveromyces lactis	NCBI
Magnaporthe grisea	NCBI
Neurospora crassa	NCBI
Phanerochaete chrysosporium	JGI
Saccharomyces bayanus	NCBI

Saccharomyces castellii	NCBI
Saccharomyces cerevisiae	NCBI
Saccharomyces kudriavzevii	NCBI
Saccharomyces mikatae	NCBI
Saccharomyces paradoxus	NCBI
Schizosaccharomyces pombe	NCBI
Ustilago maydis	NCBI
Yarrowia lipolytica	NCBI
Hexapoda	
Anopheles gambiae	NCBI
Nematoda	
Brugia malayi	TIGR
Caenorhabditis elegans	NCBI
Caenorhabditis briggsae	NCBI
Trematoda	
Schistosoma mansoni	TIGR
Capsaspora	
Capsaspora owczarzaki	PEPdb
**Amoebozoa**	
Entamoeba histolitica	TIGR
Entamoeba moshkovski	Sanger
Entamoeba invadens	TIGR
Entamoeba dispar	TIGR
**Excavates**	
Parabasalia	
Trichomonas vaginalis	TIGR
Diplomonadida	
Spironucleus barkhanus	AJR
Heterolobosea	
Naegleria gruberi	AJR
Euglenozoa	
Euglena gracilis	PEPdb
Leishmania braziliensis	Sanger

Leishmania infantum	Sanger
Leishmania major	Sanger
Trypanosoma brucei	TIGR
Trypanosoma brucei gambiense	Sanger
Trypanosoma cruzi	TIGR
Trypanosoma vivax	Sanger

Websites that were searched using BLAST are indicated. URLs for the most common genomic DNA resources are: NCBI (http://www.ncbi.nlm.nih.gov/BLAST/), TIGR (http://www.tigr.org/tdb/euk/), Sanger (http://www.sanger.ac.uk) and JGI (http://genome.jgi-psf.org/). Additionally, primers for genomic PCR were designed from unpublished EST sequence data generated under the auspices of the Protist EST Program (PEP) and from in-house EST sequencing projects in the laboratory of A. J. Roger (AJR; Dalhousie University).
