# Supplementary material for: An ancient spliceosomal intron in the ribosomal protein L7a gene (Rpl7a) of Giardia lamblia
Source: BMC Evol Biol. 2005 Aug 18;5:45. doi: 10.1186/1471-2148-5-45 (PMC1201135; doi:10.1186/1471-2148-5-45)
Supplement: Additional File 2 — L7aTree. This file (PDF format) presents a maximum likelihood tree generated using L7a sequences from representative eukaryotic and archaeal taxa. Also included are the methods used to generate the tree, the sources of the sequences and the alignment that was used. [file 1471-2148-5-45-S2.pdf]

Screening Rpl7a for Eukaryote-Eukaryote LGT

The possibility exists that the presence of the intron in the *G. lamblia* Rpl7a gene might be the result of a eukaryote-eukaryote LGT event. That such events occur, albeit rarely, seems now to be well supported and it is thus prudent to eliminate this as a potential source of contamination. Complete alignment of a representative sample of L7a sequences from 20 eukaryotes and two archaeons, followed by trimming to remove low-confidence aligned positions, left approximately 236 aligned residues. The L7a sequence is not strongly conserved, so phylogenetic noise is relatively high for this protein. The results of 100 bootstrap replicates with PROML using the JTT+Gamma model, resulted in a poorly resolved tree with strong support only for the Metazoa as a clade, as well as for one jakobid subgroup. The *G. lamblia* sequence clustered basally within the eukaryotic radiation, and specifically as a sister taxon to the *Spironucleus barkhaus* sequence, albeit at less than 50% bootstrap support. We take this to indicate that there is no strong association of the *G. lamblia* sequence with any phylogenetically inappropriate taxa, and thus no indication that this gene has entered the *G. lamblia* genome as the result of a lateral gene transfer event.

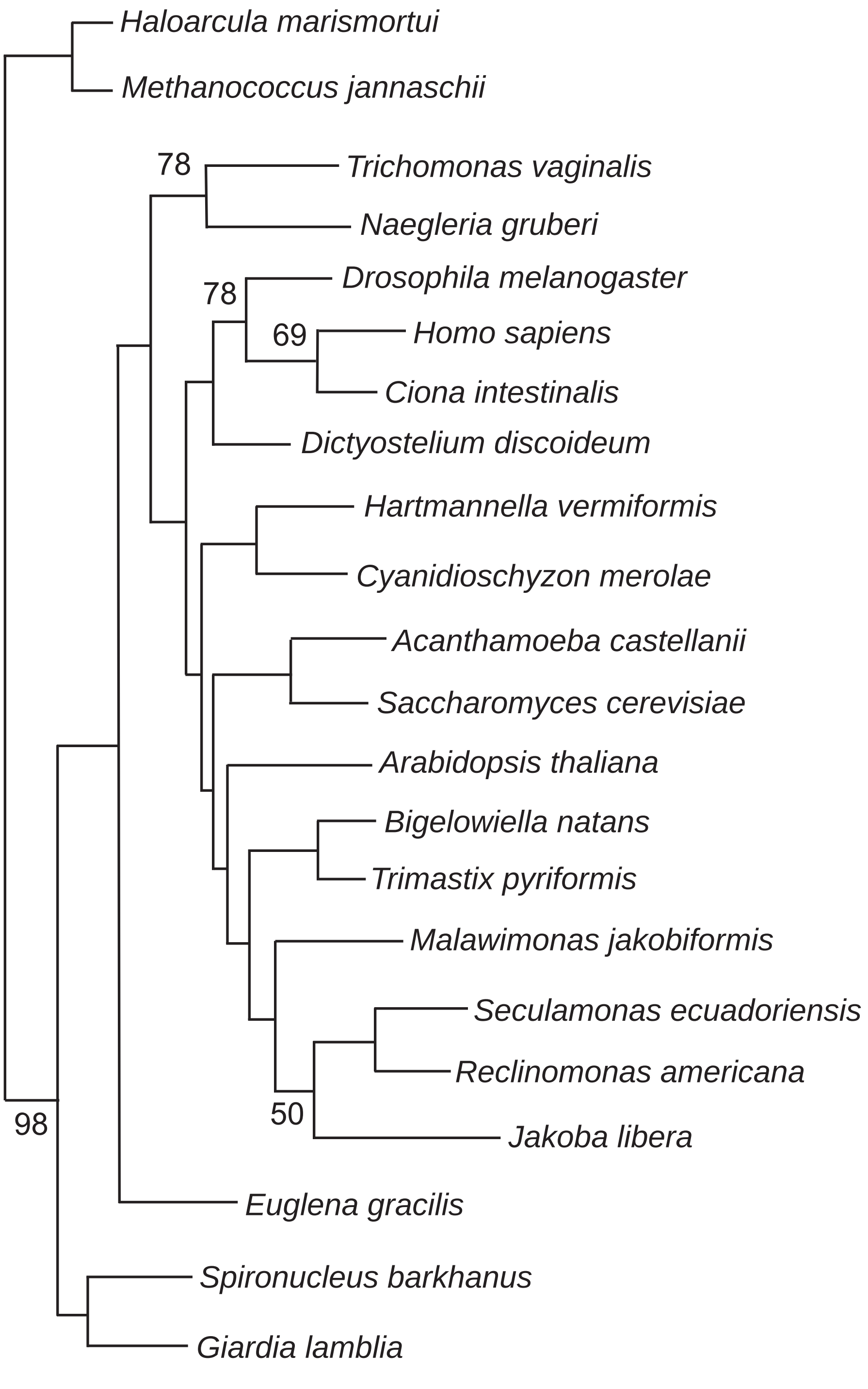

Haloarcula marismortui
Methanococcus jannaschii
78
Trichomonas vaginalis
Naegleria gruberi
Drosophila melanogaster
78
69
Homo sapiens
Ciona intestinalis
Dictyostelium discoideum
Hartmannella vermiformis
Cyanidioschyzon merolae
Acanthamoeba castellanii
Saccharomyces cerevisiae
Arabidopsis thaliana
Bigelowiella natans
Trimastix pyriformis
Malawimonas jakobiformis
Seculamonas ecuadoriensis
Reclinomonas americana
98
50
Jakoba libera
Euglena gracilis
Spironucleus barkhanus
Giardia lamblia

L7a

Accession Numbers

Rpl7a (partial EST sequences)

Acanthamoeba castellanii	AY925000
Arabidopsis thaliana	NM_130329
Bigelowiella natans	DQ118090
Ciona intestinalis	DOE Joint Genome Institute (http://genome.jgipsf.org/ciona4/ciona4.home.html) (ID:ci0100137466)
Cyanidioschyzon merolae	*Cyanidioshyzon merolae* Genome Project (http//merolae.biol.s.u-tokyo.ac.jp/) (ID:CML317C)
Dictyostelium discoideum	AC116100
Drosophila melanogaster	X82782
Euglena gracilis	AY925002
Giardia lamblia	*Giardia lamblia* Genome Database (contig 4036) (www.mbl.edu/Giardia)
Haloarcula marismortui	YP_134885
Hartmannella vermiformis	AY925001
Homo sapiens	X52130
Jakoba libera	AY924997
Malawimonas jakobiformis	AY924996
Methanococcus jannaschii	NP_248198
Naegleria gruberi	DQ118091
Reclinomonas americana	AY924999
Saccharomyces cerevisiae	AAB65045
Seculamonas ecuadoriensis	AY924998
Spironucleus barkhanus	DQ118093
Trimastix pyriformis	DQ118092
Trichomonas vaginalis	TIGR (*Trichomonas vaginalis* Genome Project) (ID: 43310.m00099)

L7a Sequence Alignment

```
22   236
Hver_L7a   QKKTNTKLFE KTPKNFGIGQ DVAPKRNMTH FVRWPKYVRL QRQHRILLNR LKVPPVINQF
Cmer_L7a   TEKASKRLFE RRPKNFGIGQ SIQPKRDLSR FVRWPRYVRL QRQRKILLQR LKVPPAIAQF
Scerevisia KKVAPAPLTH STPKNFGIGQ AVQPKRNLSR YVKWPEYVRV QRQKKILSIR LKVPPTIAQF
Acas_L7a   KQVAAAPLFE KRPKNFGIGQ DIQPKRDLTR FVRWPKYIRM QRQKRVLLHR LKVPPTINQF
Hsap_L7a   KKVAPAPLFE KRPKNFGIGQ DIQPKRDLTR FVKWPRYIRL QRQRAILYKR LKVPPAINQF
Cint_L7a   KRVAPAPLFE KRPKNFGIGQ DIQPKRDLTH FVRWPKYIRL QRQKVVLQKR LKIPPAINQF
Dmel_L7A   KKVAPAPLFE KRPKNFGIGQ NVQPKRDLSR FVRWPKYIRV QRQKAVLQKR LKVPPPIHQF
Atha_L7A   VKVA---LFE RRPKQFGIGG ALPPKKDLSR YIKWPKSIRL QRQKRILKQR LKVPPALNQF
Jlib_L7a   RVVAKPNLHV ARPKNFGLGG TVQPKRDVTR FVKWPKYIRL QRQRSILLRR LKVPPSIRQF
Secu_L7a   KPYEKPGLFP STPKTFGIGG TVLPKRDLTR FVKWPQYVRL QRQRSILLRR LKVPPAINRF
Rame_L7a   XXXXEP-LHL ARPKNMSVGA SVRKVKDLTR FVRWPKYVRL QRQRSILNRR LKVPPAINHF
Mjak_L7a   PFIPGSKLFD SRKLNFGIGQ SLKKGLDLSR YVKWPRYVRL QRQRKILYQR LKVPPSIAQF
Tpyr_L7a   KKTAGKKLIA KRPRSFHVGG DVHPPRDVSR YVKWPKYIRL QRQKKVMYQR MKTPAMINQF
Bnat_L7a   RRVAANKLFK KTPKDFRIGR SVQPKQNLSR FVKWPRYIRI QRQKAILKQR LRVPPAVNQF
Ddis_L7a   TKAAPAKLYT KNVKNFGTGF GVQPKRDLTH FTHWPRYIKL QRQRRVLLKR LKVPPTINQF
Tvag_L7a   EKVIED-LFA EETAEK---- -VADKTAQTR FPK---YVQL QRQKRILMKR LKVPPPVNHF
Ngru_L7a   KKVVKTSLLA PRPKDIGIGR DLGAKMNLTR FVKWPVYVRL QRQKRILLKR VKIPPAINQF
Egra_L7a   KDATGKKLFE SRPKNFSVGQ DLQPKRDLSR FVRWPAYIKR QRQKRILLKR LRVPPAINQF
Glamblia   SKVSGSDLAV PENKSRS--- --KCDFDLTP FVRWPRQVRI QRQKAVLQRR LKVPPTVNQF
Sbar_L7a   PNSSSSKIVK TTVKTSQMGV GVHTQKDLTR YVRWPAYIRI QRQKALLQTR LKVPGAINQF
Hmarismort XXXXXXXXXX XXXXXXXXXX XXXXXXXXXX XXXXXXXXXX XXXXXXXXXX XXXXXXXXXX
Mjannischi XXXXXXXXXX XXXXXXXXXX XXXXXXXXXX XXXXXXXXXX XXXXXXXXXX XXXXXXXXXX

           TNTLDKNAAT SLFKILHAHR PEDKASKKKR LLEAAQKAKK PK-FVKMGIN HVTSLVESKK
           QKTADKNLTD SLFRLLERYR PEEKVAKRQR LQQAAERTPK PI-FVKHGVN HVTDLIEQKK
           QYTLDRNTAA ETFKLFNKYR PETAAEKKER LTKEAAASPK PY-AVKYGLN HVVALIENKK
           TRTLDKNLAK NLFAFVDKYR PETKAEKADR LKKRAAEVAT PY-FVKYGLN HVTSLVESKK
           TQALDRQTAT QLLKLAHKYR PETKQEKKQR LLARAEKTKR PP-VLRAGVN TVTTLVENKK
           TNTLDRQTAT SLFRLAKKYQ PESKLEKRER LRQRAQETRR PL-VVSSGVN TVTNLIERKK
           SQTLDKTTAV KLFKLLEKYR PESPLAKKLR LKKIAEAKKK PS-YVSAGTN TVTKLIEQKK
           TKTLDKNLAT SLFKVLLKYR PEDKAAKKER LVKKAQASKK PI-VVKYGLN HVTYLIEQNK
           RFTLDKNVAT QLITLLSKYS PETKAEKSAR LLAAAQEKKK PF-VLKFGLN HITTLVEQKK
           SHVLDKNLAT NLFKLLIKYR PEDKQEKNAR LKAAAEATSK PY-VVKYGLN HITSLVEQKK
           TFTLNKNAAT NLFKLLLKYR PETRSEKRSR LREQAANSTK PH-FVKYGLN HIVSLVESKE
           TNALDKNNAT SLFKLLHKYR PEDKAEKAAR LKAAGEATKK PV-VLKYGLN HITSLVENNK
           TQTLDKHVAT EMFKLLHKYR PEDKKAKKER LLKLAEAPKK PM-TLKCGLN HITTLVEQKK
           TNTIDKNQAM TLFKLLAKYR PENRKEKQER LKAIAEEKDK PK-VLKYGLN HITSLVESRK
           TRVFDKNTAV HLFKLLDKYR PEEASVKKAR LLKIAEAAEK PVQHLRFGID SVTKLIEKKK
           NHTLGKDAAV ALFKFLEKYR PETKTEKKQR NKEDAEKSGN KK-ALVQGVK NVTAAIESKK
           RIVADKPLAH SVVQFLAKYK PEDEQQKKER LRQAAKDKKS ES-SLIHGIN EVVKAVERKQ
           NHTVDRHLKK ELFKFALKYK PESSFERRSR LKKEAEAVPG PR--VYSGAQ RVFRLVEQKR
           MNPISRNLTN EIFNLARKYS PESKEEHKAR LLQIADA-KS DKLVIASGIR RITSLVESKR
           HHPVSKNLNI EILNFAKKYI KETREERKVR ITKMAET--- ----VISGIN EVTNLIEKKK
           XXXXXXXXXX XMPVYVDFDV PADLEDDALE ALEVARD--- ----VKKGTN ETTKSIERGS
           XXXXXXXXXX XMAVYVKFKV PEEIQKELLD AVAKAQK--- ----IKKGAN EVTKAVERGI

           AKLVVIAHDV DPIEIVMWLP TLCVKMGIPY VIVKGKARLG QVVHKKTAAV LAVTEVDPKF
           AKLVIIAHDV DPIELVMWMP ALCRKLDIPY VIVKGKARLG ALVHLKTATC LAVTGVHDRD
           AKLVLIANDV DPIELVVFLP ALCKKMGVPY AIVKGKARLG TLVNQKTSAV AALTEVRAED
           AKLVVIAHDV DPIELVVWLP SLCKKVGVPY CIVKSKSRLG QVVHKKTSAV LAITNVRKED
           AQLVVIAHDV DPIELVVFLP ALCRKMGVPY CIIKGKARLG RLVHRKTCTT VAFTQVNSED
           AQLVVIAHDV EPVEIVVYLP ALCRKMNVPY CIVKGKSRLG RLVHRKTCTC VAITDVNNED
           AQLVVIAHDV DPLELVLFLP ALCRKMGVPY CIVKGKARLG RLVRRKTCTT LALTTVDNND
           AQLVVIAHDV DPIELVVWLP ALCRKMEVPY CIVKGKSRLG AVVHQKTASC LCLTTVKNED
           AKLVVIAHDV DPIELVLWLP ALCRKMDVPY CIIKGKSRLG QLVHQKTATC VALTDVADEH
           AKLVVIAHDV DPIELVVWLP TLCKKMGVPY CIVKGKARLG QVVHQKNATA LAFVDVRDED
           AKLVIIAHDV DPIELVVFLP VLCRRMGVPY CIVKGKARLG QVVHKKTATA LAITSVRDED
           AKLVVIAHDV DPIELVLWLP ALCRKRNVPY VIVKSKSRLG QLVHKKTATA VALTGVRAED
           AKLVVIAHDV DPIELVIWLP TLCRKMDVPY CIVKGKARLG TLVGLKTATC LALTDVKPED
           AKLVVIAHDV DPIEMVIWLP TLCRKMKVPF CIVKGKARLG ALCHMKTCTA VAITDVEKQD
           AKLVVIAHDV DPVELVLYLP TLCRRMDVPY CIVKSKSRLG ELVHMRNASC VALTGVNSAD
           AQLVIIAHDV DPIELVIWMP ALCRNLEIPY CIVKSKSRLG QIVGMKTCSC VALAEVKPED
           ASLVVIAHDV EPIELVLFLP ALCKKLDIPY VIVKSKSRLG QLVHMKNCAA VALTEVKNED
           AKLVLIAHDV DPIEIVLCLP ALCRKQGIPW CIVKGKANLG KLVGLKTATS LAFVDIKNGD
           AKLVLIANDV DPLELVLWLP TLCHKMGVPY AIVRTKGDLG KLVHLKKTTS VCFTDVNPED
           AKLVLIANDV DPIELVMWLP SLCHKMQIPY AIIRSKSELG ALAGLKTCAV LAIDEIRSED
           AELVFVAEDV QPEEIVMHIP ELADEKGVPF IFVEQQDDLG HAAGLEVGSA AAAVTDAGEA
           AKLVIIAEDV KPEEVVAHLP YLCEEKGIPY AYVASKQDLG KAAGLEVAAS SVAIINEGDA
```

```
STDFTNLVAL AKDQYNNKYT EQMKKYGGRT FGYKHTSQKA KQDRRRRKEE AKKEQX
RAELSKIIEV CKMRFNDRYE EIRRQWGGGV LGIKSTHKLE KRRRALAIEE AKRAQA
EAALAKLVST IDANFADKYD EVKKHWGGGI LGNKAQAKMD KRAKNSDSAX XXXXXX
QPALATLTKA IQENYNDRYD DLRRQWGGLQ LGRKSVHKQK AKAKAAAANQ XXXXXX
KGALAKLVEA IRTNYNDRYD EIRRHWGGNV LGPKSVARIA KLEKAKAKEL ATKLGX
KGALSKLVES VRTNYNERFD EIRRHWGGGI MGNKSLARIA KIEKLRAKDA AQKASL
KANFGKVLEA VKTNFNERHE EIRRHWGGGI LGSKSLARIS KLERAKAREL AQKQGX
KLEFSKILEA IKANFNDKYE EYRKKWGGGI MGSKSQAKTK AKERVIAKEA AQRMNX
SAAFNRLVES CKLSLPDEHX XXXXXXXXXX XXXXXXXXXX XXXXXXXXXX XXXXXX
KNSFSKLLES IENAHVENXX XXXXXXXXXX XXXXXXXXXX XXXXXXXXXX XXXXXX
VGALSRVLES VDGAIPKDSH ALNXXXXXXX XXXXXXXXXX XXXXXXXXXX XXXXXX
EHDFAQLVQV ARTSYXXXXX XXXXXXXXXX XXXXXXXXXX XXXXXXXXXX XXXXXX
KKTFSDLVAV CRTNYIDRAE AIVRQQGGGK LGVKSAKKIE KRQKALAKDE KQREKM
DRALNQFVAS VAPMYEEAPR AWGAVG---- FGIKSRHKIR ARQXXXXXXX XXXXXX
SNELALLVES AKQMFNNN-S EHRKTWGGNT LSGPARAILA KRQKAEAKES LAKSKX
RAAFTKIVDS VNSGFLAHYK EEMHQWGGGE LSEKTIEKLK AQGKYKEXXX XXXXXX
RDPFAKIVEA VRGAFVDRFR TVNTKWGGGQ LSKRSIIEQK NRIKKLKXXX XXXXXX
KTDFEKLTQS VKLAYNDKYE ELSRKWGGLR LSKKSKQKMA KKKRIAAANA AKXXXX
KPTFDKILAA V--AHEVDYA KAMKTYGGGV RREDEAQQMX XXXXXXXXXX XXXXXX
TAALRSITDK IAVEVN--YE KTIKNHGGNT LSLRSQRKLP SVEKHQFYQF EKKKKK
DADVEDIADK VEELRXXXXX XXXXXXXXXX XXXXXXXXXX XXXXXXXXXX XXXXXX
E-ELKVLIEK VNVLKQXXXX XXXXXXXXXX XXXXXXXXXX XXXXXXXXXX XXXXXX
```
